# The lexicalization of emojis: the influence of frequency and functions of emojis in sentences on this process—a study based on eye movement tracking

**DOI:** 10.3389/fpsyg.2025.1631967

**Published:** 2025-09-17

**Authors:** Wanhong Lu, Haoge Du, Feng Gu, Jianghua Han

**Affiliations:** ^1^Neurocognitive Laboratory for Linguistics and Semiotics, College of Literature and Journalism, Sichuan University, Chengdu, China,; ^2^Digital Convergence Laboratory of Chinese Cultural Inheritance and Global Communication, Sichuan University, Chengdu, China

**Keywords:** emojis, lexicalization, frequency, eye tracking, function of emojis

## Abstract

The lexicalization of emojis reflects the dynamic evolutionary characteristics of the linguistic symbol system in the digital age. The influence of usage frequency and the different functions of emojis in sentences on this process is also a research topic worthy of exploration. This study employed eye-tracking technology, with 98 native Chinese speakers as participants, and selected Chinese sentences as experimental stimuli to compare the processing differences of emojis with different frequencies (high frequency and low frequency) and different functions in sentences (Pro-text emojis, Co-text emojis, and words) during sentence reading. The research results show that: Significantly affects the first fixation duration and total fixation duration. High frequency emojis have shorter durations for these two indicators; in contrast, low-frequency emojis require more time for recognition and integration due to visual and semantic factors. Pro-text emojis have a longer fixation duration, while Co-text emojis have a shorter total fixation duration. In the integration stage, Pro-text emojis take longer to integrate. This difference is related to the unique cognitive pattern of emojis, which requires converting images into linguistic components before integrating them into sentences for comprehension. Co-text emojis, on the other hand, take less time, which may be attributed to the priming effect triggered by the text preceding Co-text emojis. There is no significant difference in the number of saccades between emojis and Chinese text, indicating certain similarities between the two. In conclusion, lexicalized Pro-text emojis can be integrated into daily language communication; high frequency emojis have greater advantages in lexical recognition and processing; different functions of emojis in sentences affect their roles in text and processing mechanisms. Conducting research with Chinese as the experimental material provides a new perspective for the study of emoji processing.

## 1 Introduction

Inserting emojis into online chat texts has become a prevalent form of expression in the digital age, seamlessly integrating into people’s daily lives (Holler and Levinson, 2019). Initially, emojis were employed to replicate facial expressions in written communication. This allowed users to infuse non-verbal emotional cues into online texts, thereby conveying information in a more vivid and precise manner (Neel et al., 2023; Rodrigues et al., 2022). They not only capture the subtleties of the sender’s emotions but also offer crucial context clues, enabling recipients to accurately interpret the sender’s intentions (Prada et al., 2018).

In online chats, there are examples of the interactive integration of “text-emoji” expressions. The “text-emoji” interaction refers to a form of communication in digital communication scenarios (such as online chats, social media interactions, etc.) where two information transmission modalities—written text and emojis—cooperate with and synergize with each other. In most “text-emoji” interactions, the text and emojis are integrated through a shared conceptual framework, effectively communicating comprehensive meanings. This is exemplified by processes like image integration and gesture integration (Ganis et al., 1996; Cohn, 2016). Recent research has demonstrated that this multi-modal integration tested using gestures, animations, and images can prompt observers to draw semantic and pragmatic inferences from pictures or gestures that are as reliable as those derived from standard words (Hintz et al., 2023; Pérez et al., 2020; Schlenker, 2019; Tieu et al., 2019). Specifically, Hancock et al. (2024) found that emojis, functioning as gestures in digital communication, enhance the comprehension of indirect speech, further validating the pragmatic role of emojis in multi-modal meaning construction. Beyond semantics and pragmatics, scholars exploring the multi-modal integration model have also examined the syntactic interplay between emojis and written text. Emojis convey concepts in the same way as words, suggesting that emojis have been assimilated into the overarching communication system rather than functioning as an isolated entity separate from text (Cohn and Schilperoord, 2022). Under the framework of the multi-modal integration model, emojis serve dual functions: they not only convey semantic information within sentence level contexts but also play a role in facilitating reading comprehension (Lo, 2008).

Storment (2024) verified, using a large number of examples in English, German, and Spanish, that some emojis can actually appear as contentful morphological units that behave according to regularly predictable morphosyntactic rules. He classifies these emojis according to their linguistic functions in sentences. From a semantic perspective, and by drawing on terminology from the field of paralinguistics and the terminological system of gesture semantics research (Schlenker, 2019), he divides the positions of emojis into three main categories: “Post-text emojis,” “Co-text emojis,” and “Pro-text emojis.”In example (1),the emoji serves as “Co-text emojis,” where emojis directly follow without spacing a word or phrase that they modify. Pro-text emojis are a category of emojis that possess specific syntactic properties, have a clear etymology of their name, and enjoy a wide range of usage, as illustrated in example (2). Among these, Pro-text emojis specifically refer to those semantic projections directly embedded after specific words or components, such emojis are syntactically independent and belong to lexicalized emojis.

1. I can build a house 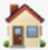 rebuild a car 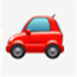 cheand dig your grave!!! (Storment, 2024)
2. She is the 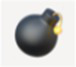 [int: bomb] (Pierini, 2021)

Earlier studies primarily focused on facial emojis and their role in conveying emotions within discursive contexts. In recent years, emoji research has expanded to examine semantic integration with text and neural response disparities between emojis and words in multimodal processing frameworks. For example, Ousterhout (2017) investigated the semantic priming effects of symbolic pictures (e.g., emojis) in text using event-related potential (ERP) components, specifically the N400 (The N400 is a negative-going event-related potential (ERP) component that typically emerges approximately 400 milliseconds after participants are exposed to linguistically semantically inconsistent, implausible, or unexpected stimuli). By comparing N400 amplitudes between symbolic pictures and words in priming tasks—and observing amplitude differences under semantically related vs. Unrelated conditions (e.g., larger N400 for unrelated stimuli)—the study tested whether emojis share neural mechanisms with words. ERP results revealed that symbolic pictures exhibit N400 effects akin to words during semantic processing, supporting the lexicalization hypothesis and providing empirical evidence for symbol-text integration in multimodal communication.

A comprehensive review of the literature on the electrophysiological responses to Co-text emojis and Pro-text emojis reveals that emojis evoke neural patterns that share significant similarities with those evoked by words. Using electroencephalography (EEG), another study examined whether Co-text emojis could evoke incongruity effects (i.e., N400), but findings were inconclusive. The author hypothesized that inconsistent participant interpretations of experimental emojis—owing to their semantic ambiguity—might have confounded results. In a Chinese-language study, semantically inconsistent words triggered robust N400 and P600 effects, whereas Co-text emojis elicited only a prominent and prolonged N400, suggesting emojis pose greater semantic retrieval challenges and lower integration efficiency within contexts. Collectively, these results indicate that emoji semantic processing in sentential contexts differs from word processing, with distinct challenges at the sentence level (Tang et al., 2020). However, the impact of varying functions of emojis on these processing differences remains underexplored.

A study by Robus et al. (2020) utilized an eye tracking paradigm to investigate Co-text emoji processing. They found no significant emotion-induced effects in neutral sentences. Emojis Co-text emojis at sentence-final positions show longer reading times and fixation durations than initial positions, attributed to wrap-up effects during semantic integration. Barach et al. (2021) found that Co-text emojis (non-facial emojis) which are semantically congruent with the meaning of the complex sentence they are in have shorter fixation durations and lower refixation frequencies than those that are semantically incongruent. Additionally, emojis that align with the meaning of the complex sentence are skipped more frequently—which suggests that, similar to words, emojis can convey semantic information through parafoveal preview. Christofalos et al. (2022) showed that sentences with synonymous or inferentially consistent Co-text emojis exhibit higher perceived coherence and better emoji recall, indicating seamless integration of such symbols into discourse memory representations. Although these studies address emoji contributions to discourse meaning and processing, the phenomenon of lexicalization remains unaddressed.

Despite facial emojis comprising a large share of overall usage, noun and adjective Pro-text emoji emojis is more common due to these word classes’ clearer semantic boundaries. Research shows Pro-text emojis for nouns/adjectives integrate effectively into sentence structures, with semantically specific emojis favoring preferred grammatical positions. Cohn et al. (2018) found Pro-text emojis deviating from optimal grammatical positions increase subsequent word processing costs. Cohn et al. (2019) further noted that while Pro-text emojis convey meaning, their linear grammatical structure limits them primarily to noun or adjective roles rather than verbs or adverbs. Weissman (2019) revealed that emojis (e.g., food, animals) in sentences evoke neural responses (late frontal positivity, N400) similar to semantic violations by words, suggesting Pro-text emojis generate word-like semantic violation effects. However, Paggio and Tse (2022) demonstrated processing costs in eye-tracking metrics for Pro-text emojis, proposing Pro-text emojis tokens are less seamlessly integrated than words. Notably, extended fixation times may reflect general cognitive mode shifts rather than intrinsic emoji processing costs.

Previous research on emoji processing has predominantly relied on methods such as self reporting (e.g., Kelly and Watts, 2015), the self- paced reading paradigm (e.g., Cohn et al., 2018), the modified rapid serial visual presentation (RSVP) paradigm (e.g., Weissman, 2019), and overall reading time measurements (e.g., Gustafsson, 2017). In contrast, the present study aims to provide a more fine grained analysis by examining both the early and late stages of eye movement measurements during emoji processing, thereby obtaining detailed temporal information.

This study has a significant difference from previous studies that used alphabetic scripts such as English as experimental materials. This study used Chinese as the experimental material. During reading, Chinese texts exhibit a more pronounced semantic preview advantage compared to texts in alphabetic languages (Yan et al., 2009; Yang et al., 2012). Schotter et al. (2012) posited that the more compact spatial arrangement of Chinese words, as opposed to those in alphabetic languages, allows for more upcoming words to be positioned closer to the fovea, thereby enhancing foveal processing. Similar to Chinese characters, emojis convey rich semantic information through a compact spatial layout. Spinks et al. (2001) found that ideographic scripts, compared to phonetic scripts, demand longer fixation times, and the right hemisphere of the brain is more engaged during reading. Both emojis and Chinese characters are character based. Emojis are pictographic in their written form; Chinese characters, as ideograms, carry information related to form, sound, and meaning. Research by Scheffler et al. (2022) indicates that although emojis lack standard pronunciations, they can activate the entire lexical entry, including phonetic information, when the context is semantically consistent.

The core of lexicalization lies in the process where non-lexical units gradually acquire fixed semantic meanings, syntactic functions, and the ability to be used independently, eventually becoming linguistic elements similar to words. High frequency usage strengthens the semantic fixity of emojis and facilitates the transformation of specific emojis into Pro-text emojis. Based on this, and with reference to Storment (2024), which uses linguistic theories to demonstrate Pro-text emojis (high frequency emojis have acquired independent meanings and can function as lexical items). We hypothesize that Pro- text emojis are lexicalized emojis, that means high frequency Pro-text emojis can be comprehended in the same way as standard words, and this study will adopt experimental methods to verify this claim. Additionally, it will explore how the linguistic functions of emojis in sentences and their usage frequency influence the processing of emojis and words.

## 2 Methodology

### 2.1 Power analysis

In the realm of research design, power analysis serves as a fundamental aspect. The present investigation utilized G*power 3.1 software to ascertain the requisite sample size, a crucial step in ensuring the statistical validity of the research. A medium level effect size of 0.25 for repeated measures was set in this study, and the α value was fixed at 0.05. The calculation results demonstrated that to attain a statistical power of 0.95, the study necessitated at least 66 participants. To guarantee sufficient statistical power and account for potential dropout and data corruption, 98 participants were actually recruited.

### 2.2 Participants

The participants in this experiment were 98 native Chinese speakers from Sichuan University, with 26 males and 72 females. Their mean age was 20.78 years (SD = 2.29). All participants had normal or corrected to normal vision, were free from color blindness and astigmatism (or had only mild astigmatism), and had no history of neurological or mental disorders. They were also free from reading disabilities and participated in the experiment voluntarily. Prior to the experiment, the participants read and signed the informed consent form and received appropriate compensation post experiment. We ensured that all participants used iPhone and were familiar with the emojis in the Apple iOS system.

### 2.3 Procedure

The participants were instructed to adjust the distance between their eyes and the eye tracker to approximately 65 cm. They were required to maintain a stable head position. Thereafter, the eye movement calibration of the participants was conducted. The calibration employed a five point method, and if the error was less than 0.5°, they could proceed to the next stage of the experiment. After successful calibration, the participants underwent three experimental test trials to simulate the formal experimental process.

During the experiment, a “+” was presented on the screen in front of the participants, positioned at the location of the first Chinese character of the target sentence. The participants were required to fixate on this “+,” and after 500 ms, the target sentence would be displayed. They were instructed to read the target sentence silently and press the space to indicate that they had finished reading. Subsequently, the next crosshair would appear at the start of the next target sentence. This approach was implemented to ensure that the participants initiated the reading of each target sentence from the first Chinese character.

To ensure the participants’ attentiveness and the authenticity and reliability of the experimental data, when the experiment reached the 25% mark, the participants were requested to verbally repeat the sentence they had just read. Generally, most participants could easily reproduce the sentence, although a small number might forget the initial demonstrative pronoun. Given the short duration of this experiment, approximately 20–25 min, to ensure the smooth progression of the experiment and the participants’ concentration, and considering that the Tobii eye tracker permits minor head movements, re-calibration was not required after the participants’ vocal repetition.

### 2.4 Apparatus

The Tobii Spectrum eye tracker, manufactured by Tobii Technology in Sweden, was utilized in this study. This device captures eye movement data based on the corneal reflection principle, featuring a sampling frequency of 1,200 Hz, an accuracy of 0.5°, and a drift error of less than 0.3°. The experiment was conducted in the Digital Integration Laboratory for Chinese Culture Inheritance and Global Communication at Sichuan University. This laboratory offers excellent light proof and sound proof properties, with the temperature and humidity regulated to optimal levels. The eye tracker was connected to a 23.8 inch monitor with a resolution of 1,920 × 1,080 pixels and was appropriately adjusted to ensure the precise presentation of image sizes. The brightness and contrast of the monitor were set above the optimal thresholds, and the color temperature was set to 5,800°K to provide an optimal visual experience.

### 2.5 Experiment materials

This study aimed to explore the processing disparities of Emoji emojis with varying frequencies (high frequency and low frequency) and different functions of emojis (word, Co-text emoji and Pro-text emoji) in the context of Chinese sentence processing through the analysis of eye tracking data. In this way, we aim to explore the process of emoji and the influencing factors during this process. Ten high frequency and 10 low frequency emojis were selected, with their images and usage frequencies sourced from Emojipedia.org. To mitigate any potential interference, the selected emojis contained no text or expression elements. The experimental materials were carefully chosen to be unambiguous, with each emoji representing a noun like entity and having a unique and well defined meaning.

**Figure 1:**
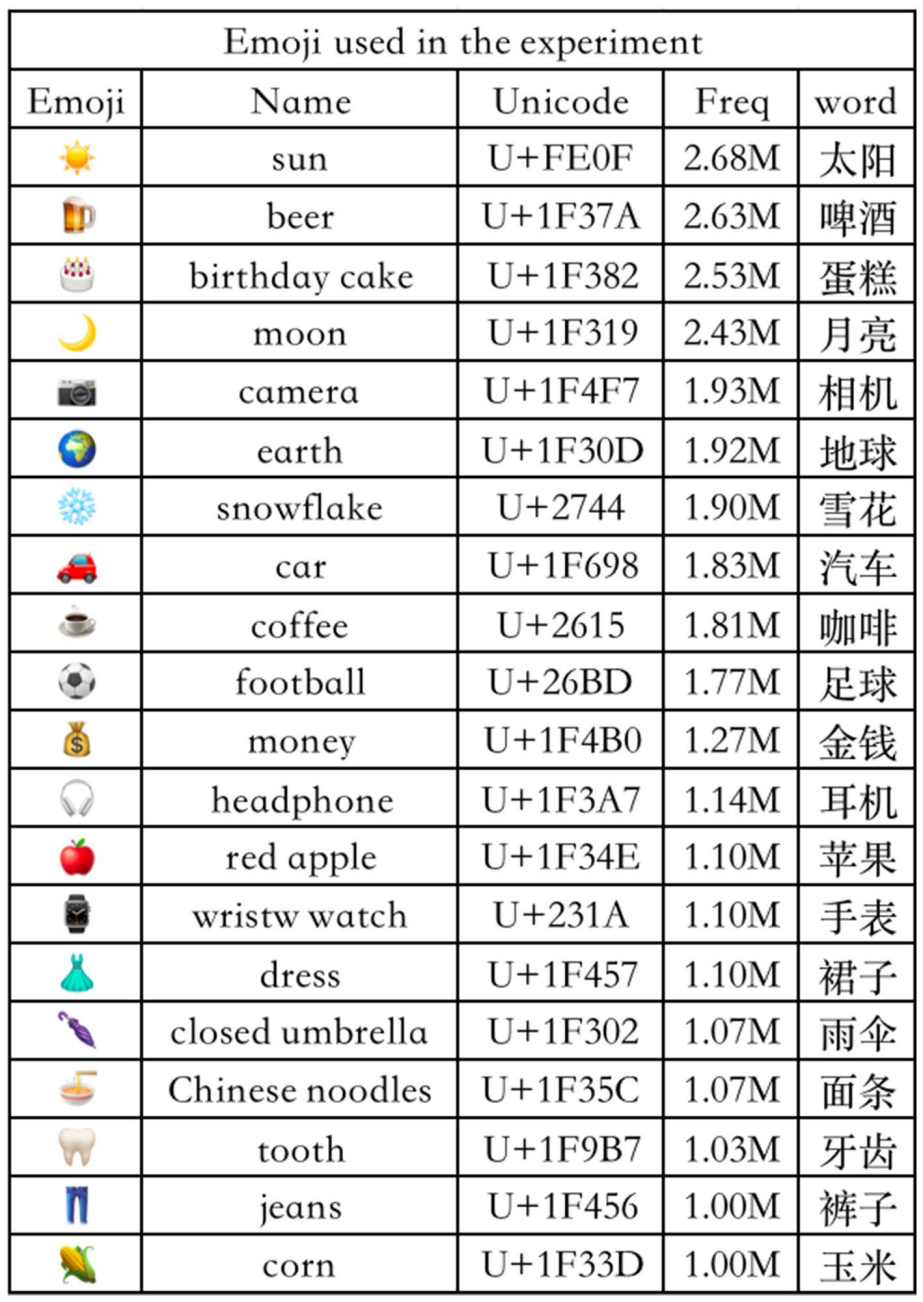
Details of emoji stimuli, including name, unicode, usage frequency, and corresponding chinese lexical equivalents.

Prior to the experiment, to validate the accuracy of the experimental material selection, we designed a questionnaire (see Appendix 1) to gather feedback from the participants. The frequency distribution and semantic correspondence of selected emojis were validated by participant questionnaires, with detailed stimuli information (name, Unicode, frequency, and corresponding Chinese words) shown in Figure 1.

In the preparation of experimental materials, we leveraged the research findings of predecessors (Scott et al., 2009; Robus et al., 2020) to design 20 distinct neutral contexts. The target sentences employed in the experiment adhered to the syntactic structure of “This/That + classifier + target word + very + adjective.” Meticulously, we ensured that the target word was not positioned at the end of the sentence to preclude additional reading time engendered by end of sentence processing. In an effort to enhance the measurement precision, we meticulously delineated the area of interest for each target word and target emoji.

In experiment, each participant randomly read 20 target sentences and 20 filler sentences. The experimental materials were arranged according to a Latin square and divided into three versions. Filler sentences, while structurally similar with a length of 8 characters, contained no target emojis and used unrelated common Chinese words to prevent participants from detecting the experimental pattern. Both target and filler sentences were balanced in terms of lexical frequency (referring to the CCL corpus) and syntactic complexity. The specific formats of target sentences based on the combination of emoji frequency and function of emojis are shown in Table 1.

**Table 1:** Target sentences with examples.

Emoji frequency	Function of emojis	Chinese sentence	English meaning
High	text	那枚戒指很精致	That ring is very delicate
	Pro-text emoji	那枚 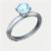 很精致	That 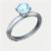 is very delicate
	Co-text emoji	那枚戒指 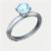 很精致	That ring 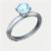 is very delicateding
Low	text	这款耳机很实用	This headphone is very practical
	Pro-text emoji	这款 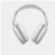 很实用	This 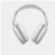 is very practical
	Co-text emoji	这款耳机 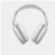 很实用	This headphone 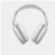 is very practical

## 3 Results

**Figure 2:**
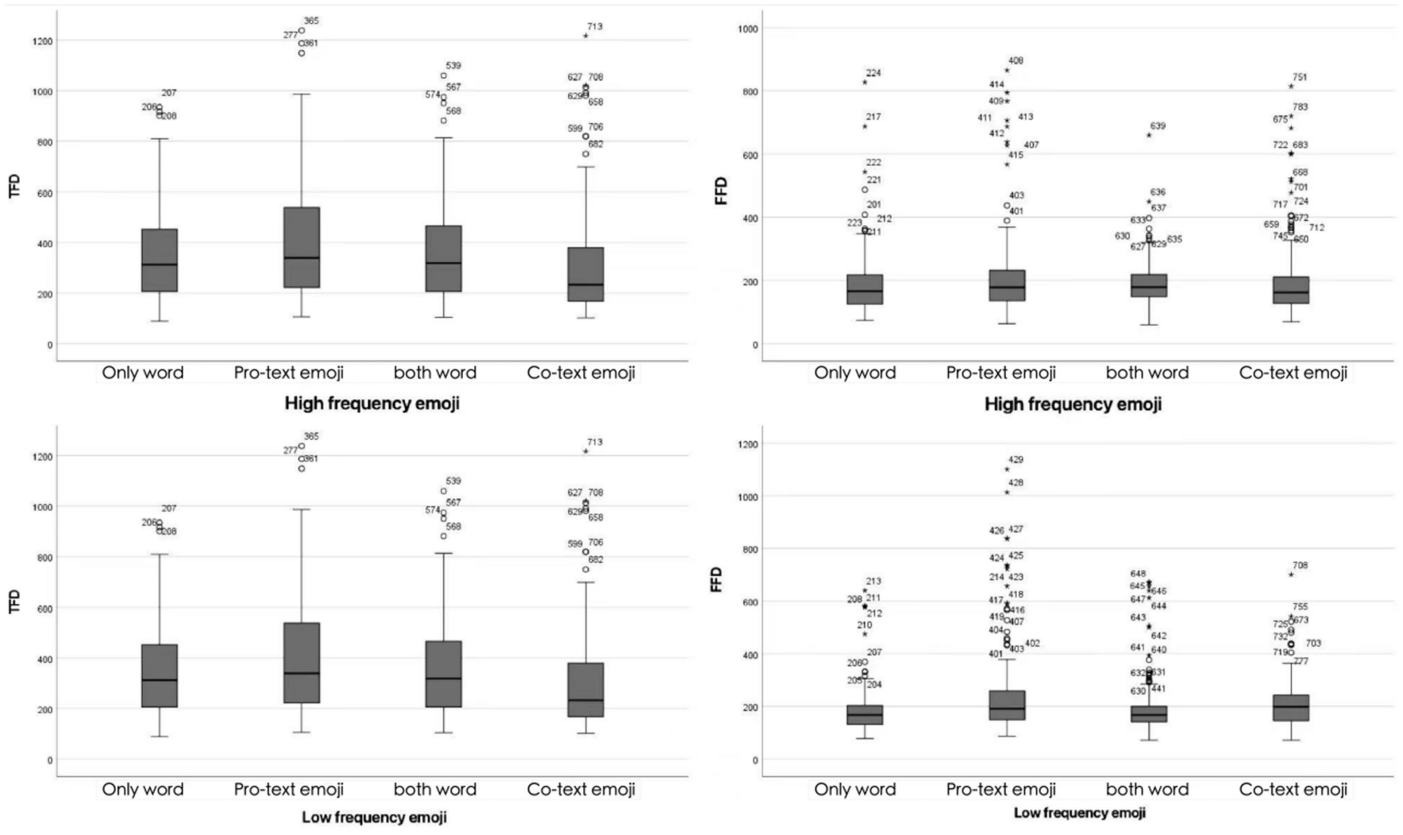
Total fixation duration (TFD) and first fixation duration (FFD) under different emoji frequencies (high/low) and usage conditions (only word, both word, co-text emojis, pro-text emojis).

In this section, we first report the results obtained from the three groups of stimuli separately, and then conduct comparisons among them. Key eye movement metrics, including total fixation duration, first fixation duration, and the number of saccades in the area of interest (AOI), were selected for analysis. A total of 3,864 data samples were collected. Values less than 100 ms and null values were ignored. Ultimately, a total of 493 data sets were excluded (accounting for 12.75% of the total observations of the three measurements). Put differently, Figure 2 all distributions display positive skewness, with outliers in the right hand tails constituting 3.34% of the total TFD distribution, 5.49% of the overall FFD distribution, and 6% of the entire Number of Saccades in the Area of Interest distribution.

In light of these findings, logarithmic transformations were meticulously applied to the values of 3,371 relevant metrics. This transformation was implemented to effectively reduce the positive skewness and ensure a more accurate representation of the data. Subsequently, For inferential statistics, the lmer Test package in R software (Version 4.3.0) was used to conduct a linear mixed-effects model analysis. These models were designed to comprehensively assess the impacts of random effects on each variable under investigation. In these models, the usage conditions and emoji frequencies were designated as fixed effects, while participants and items were classified as random effects. Present the back-transformed values in milliseconds (ms) and corresponding confidence intervals (CI) in Table 2 for interpretation.

**Table 2:** Mean, standard deviation, and 95% confidence interval values of first fixation duration, total fixation duration for different functions and targets in the experiment (ms), and Number of saccades in AOI for different frequency and conditions.

Measure	Frequency	Function	Target	M	SD	CI
TFD	High	Only word	Word	355.32	224.964	319.27,391.37
		Pro-text emoji	Emoji	407.67	237.058	370.89,444.45
		Both	Word	356.35	185.453	331.54,381.16
		Co-text emoji	Emoji	301.58	202.512	269.12,334.03
	Low	Only word	Word	359.8	203.201	331.89,387.71
		Pro-text emoji	Emoji	594.34	241.92	536.98,651.71
		Both	Word	361	232.912	347.76,410.23
		Co-text emoji	Emoji	330.78	201.782	290.81,370.74
FFD	High	Only word	Word	182.99	92.297	170.83,195.14
		Pro-text emoji	Emoji	201.59	124.221	183.82,219.37
		Both	Word	187.13	69.502	177.98,196.28
		Co-text emoji	Emoji	198.25	125.668	177.90,218.59
	Low	Only word	Word	186.58	90.462	174.39,198.77
		Pro-text emoji	Emoji	231.17	148.634	211.24,251.11
		Both	Word	187.33	95.316	174.64,200.03
		Co-text emoji	Emoji	211.69	95.384	196.19,227.18
NSA	High	Only word	Word	0.31	0.522	0.24,0.38
		Pro-text emoji	Emoji	0.25	0.44	0.18,0.31
		Both	Word	0.34	0.494	0.28,0.41
		Co-text emoji	Emoji	0.33	0.476	0.24,0.44
	Low	Only word	Word	0.34	0.476	0.28,0.41
		Pro-text emoji	Emoji	0.11	0.316	0.07,0.15
		Both	Word	0.26	0.545	0.19,0.34
		Co-text emoji	Emoji	0.24	0.555	0.17,0.34

The conclusive results are presented in Table 3, for examining the significance of various effects, including both main effects and interaction effects, within the linear mixed model framework on the dependent variables. In the subsequent sections, each eye tracking measurement index will be discussed in isolation to provide a more in depth understanding of the experimental outcomes.

**Table 3:** Summary of linear mixed effects models for total fixation duration (TFD), first fixation duration (FFD), and number of saccades in AOI (NSA), including fixed/random effects and significance tests.

	TFD	FFD	NSA
Fixed effects			
Intercept	6.938	5.6084	0.34
Condition	2.5066	2.1048	0.052
Emoji frequency	1.3879	1.2086	0.986
Emoji frequency: condition	2.7907	2.3873	2.949
Random effects			
Item intercept	0.052	0.003	0.0304
Subj intercept	0.041	0.013	0.089
Residual	0.707	0.309	0.04
X^2^	52.341	24.432	0.257
df	1	1	1
*p*	<0.001	<0.001	0.079

### 3.1 Total fixation duration results

Here, we first consider the TFD, which represents the sum of all fixation times within the AOI of the target under each condition. Based on the distributions presented in Table 2, we observed that for a word target, the TFD remains nearly identical whether the word is used alone or followed by an emoji. However, for an emoji target, for Pro-text emojis, the distribution of its TFD is longer, and the degree of variation is significantly greater compared to Co-text emojis. The total fixation duration of high frequency emojis is also shorter than that of low frequency emojis. The linear mixed effects model predicting TFD revealed a significant interaction effect between emoji frequency and function condition (X^2^ = 52.341, *p* < 0.001; see Table 3).

Pairwise comparisons of TFD across frequency and function condition levels (reported in Table 4) showed that high frequency emojis in the “Co-text emojis” condition had significantly shorter TFD than low frequency emojis in the “Pro-text emojis” condition (*p* < 0.0001). In Table 4，Simple effect analysis showed that at high emoji frequency,the dependent variable for “Co-text emojis” was 54.77 lower than that for “both word” on average. With a *t* = −9.273 and *p* < 0.0001, the difference between these two groups was significant. For “both word” and “only word” at high emoji frequency, the *t* = 0.175, and *p* = 0.9981, indicating no significant difference. At low emoji frequency, the estimated difference between “Co-text emojis” and “Pro-text emojis” was −263.57, with a *t* = −44.626 and *p* < 0.0001, showing a significant difference between the two groups.

**Table 4:** Pairwise comparisons of total fixation duration (TFD) across emoji frequency (high/low) and usage conditions (only word, both word, co-text emojis, pro-text emojis).

Emoji frequency	Contrast	Estimate	SE	df	t.ratio	*p*.value
High	Co-text emoji-both word	−54.77	5.91	63	−9.273	<0.0001***
	Co-text emoji-Pro-text emoji	−105.8	5.91	63	−17.914	<0.0001***
	Co-text emoji-only word	−53.73	5.91	63	−9.098	<0.0001***
	Both word-Pro-text emoji	−51.03	5.91	63	−8.64	<.00001***
	Both word-only word	1.04	5.91	63	0.175	0.9981
	Pro-text emoji-only word	52.07	5.91	63	8.816	<0.0001***
Low	Co-text emoji-both word	−48.22	5.91	63	−8.165	<0.0001***
	Co-text emoji- Pro-text emoji	−263.57	5.91	63	−44.626	<0.0001***
	Co-text emoji-only word	−29.02	5.91	63	−4.913	<0.0001***
	Both word-Pro-text emoji	−215.35	5.91	63	−36.461	<0.0001***
	Both word-only word	19.2	5.91	63	3.252	0.0097
	Pro-text emoji-only word	234.55	5.91	63	39.712	<0.0001***
Function						
Only word	High-low	−4.48	5.91	63	−0.758	0.4513
Pro-text emoji	High-low	−186.96	5.91	63	−31.655	<.0001***
Both word	High-low	−22.64	5.91	63	−3.834	0.0003
Co-text emoji	High-low	−29.19	5.91	63	−4.943	<.0001***

Simple effect analysis revealed significant differences in the dependent variable’s mean values across high and low emoji frequency levels for different target conditions. When targets were “Co-text emojis” the estimated difference (“high-low”) was −29.19 (*t* = −4.943, *p* < 0.0001). For “both word” targets, the difference was −22.64 (*t* = −3.834, *p* = 0.0003). With “Pro-text emojis” targets, the difference was −186.96 (*t* = −31.655, *p* < 0.0001). However, when targets were “only word,” the estimated difference of-4.48 (*t* = −0.758, *p* = 0.4513) indicated no significant difference between high and low emoji frequency levels.

### 3.2 First fixation duration results

In Table 3, the *p*-value of the interaction effect under FFD is less than 0.01, indicates a significant interaction between emoji frequency and targets. Pairwise comparisons of FFD (see Table 5) indicated that under low frequency, “Co-text emojis” conditions had significantly longer FFD than “both word” conditions (*t* = 4.292, *p* = 0.0004). We conducted between group contrast tests on FFD, and adjusted the *p*-values of multiple comparisons. The results of pairwise comparisons of each level of targets at different levels of emoji frequency, as well as pairwise comparisons of each level of emoji frequency at different levels of targets, are presented.

**Table 5:** Pairwise comparisons of first fixation duration (FFD) across emoji frequency (high/low) and usage conditions (only word, both word, co-text emojis, pro-text emojis).

Emoji frequency	Contrast	Estimate	SE	df	*t*.ratio	*p*.value
High	Co-text emoji-both word	11.118	5.68	63	1.959	0.2147
	Co-text emoji-pro-text emoji	−3.336	5.68	63	−0.588	0.9354
	Co-text emoji -only word	15.259	5.68	63	2.688	0.0443
	Both word-pro-text emoji	−14.454	5.68	63	−2.546	0.0625
	Both word-only word	4.141	5.68	63	0.729	0.8849
	Pro-text emoji-only word	18.595	5.68	63	3.276	0.0091
Low	Co-text emoji-both word	24.365	5.68	63	4.292	0.0004
	Co-text emoji-pro-text emoji	−19.476	5.68	63	−3.431	0.0057
	Co-text emoji -only word	25.111	5.68	63	4.424	0.0002
	Both word-pro-text emoji	−43.842	5.68	63	−7.723	<0.0001***
	Both word-only word	0.746	5.68	63	0.131	0.9992
	Pro-text emoji-only word	44.587	5.68	63	0.131	<0.0001***
Function						
Only word	High-low	−3.591	5.68	63	−0.633	0.5293
Pro-text emoji	High-low	−29.584	5.68	63	−5.212	<0.0001***
Both word	High-low	−0.196	5.68	63	−0.035	0.9726
Co-text emoji	High-low	−13.443	5.68	63	−2.368	0.0210

Simple effect analysis showed that under the condition of high frequency, the estimated difference between “Co-text emojis” and “both word” was 11.118 (*t* = 1.959, *p* = 0.2147), indicating no significant difference. The differences between “Co-text emojis-only word” and “Pro-text emojis-only word” were 15.259 (*t* = 2.688, *p* = 0.0443) and 18.595 (*t* = 3.276, *p* = 0.0091), both showing significant differences. And at low emoji frequency, the difference between “Co-text emojis” and “both word” was 24.365 (*t* = 4.292, *p* = 0.0004), a significant result. The differences between “both word-Pro-text emojis” and “Pro-text emojis-only word” were −43.842 (*t* = −7.723, *p* < 0.0001) and 44.587 (*t* = 7.855, *p* < 0.0001) respectively, both showing extremely significant differences.

Simple effect analysis, with the targets variable fixed, compared high and low levels of emoji frequency. When targets were Co-text emojis, the estimated “high-low” difference of the dependent variable was −13.443 (*t* = −2.368, *p* = 0.0210), showing a significant impact of emoji frequency level. When targets were Pro-text emojis, the “high-low” difference was −29.584 (*t* = −5.212, *p* < 0.0001), indicating an extremely significant impact. In contrast, when targets were both word, the “high-low” difference was −0.196 (*t* = −0.035, *p* = 0.9726), and when targets were only word, it was −3.591 (*t* = −0.633, *p* = 0.5293), both showing no significant impact of emoji frequency level on the dependent variable.

### 3.3 Number of saccades in AOI results

**Figure 3:**
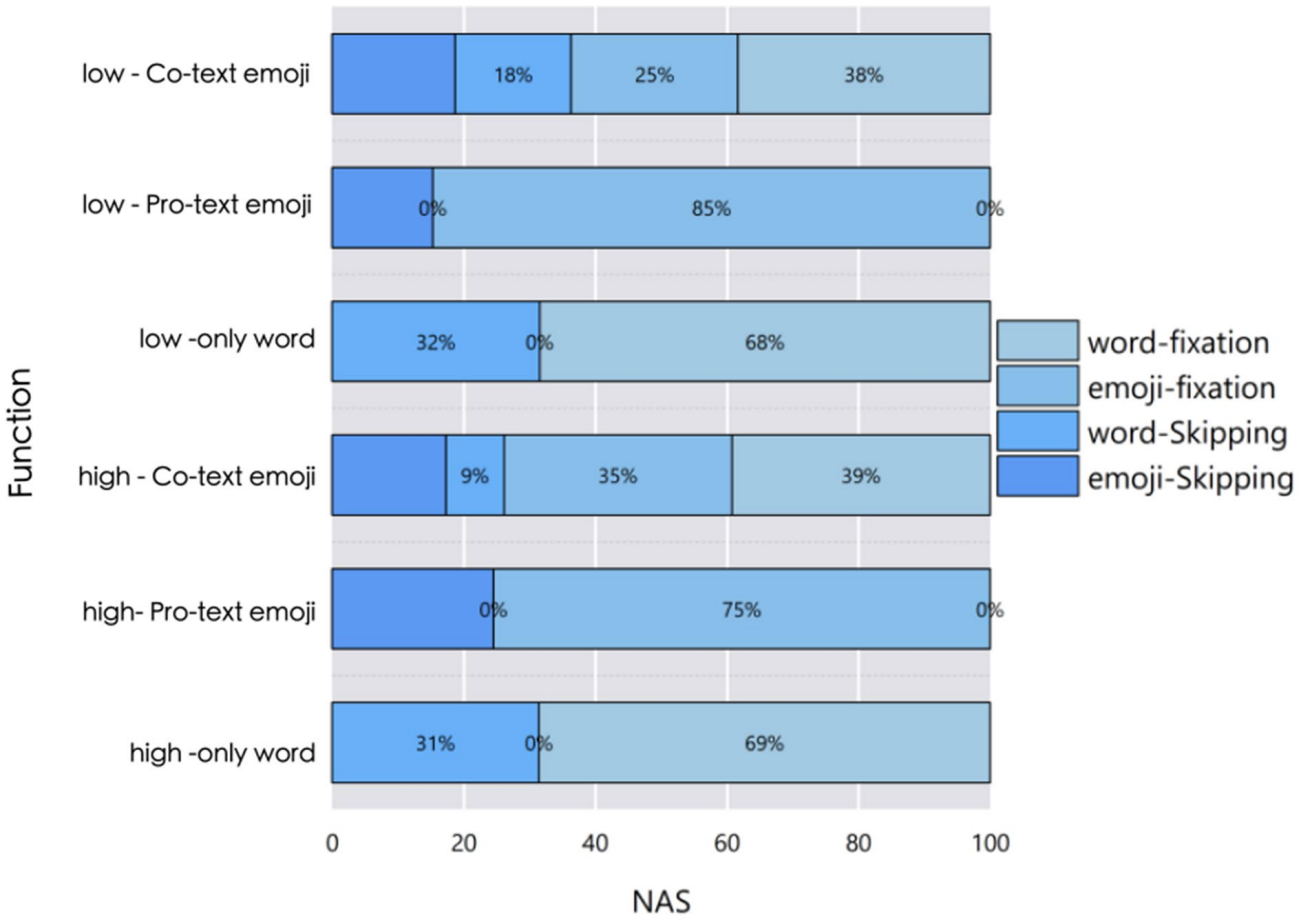
Percentage distribution of saccade behaviors (word fixation, emoji fixation, word skipping, emoji skipping) across frequencies and conditions.

Generally, words induce fewer saccades. The distribution of saccade behaviors across conditions is visualized in Figure 3, which shows that “Pro-text emojis” conditions (especially low-frequency) had higher emoji fixation rates (85% for low-only emoji) than text conditions. Regardless of the function conditions, under the condition of low frequency Pro-text emojis, the average number of fixations within the area of interest is slightly lower than in all other conditions. Overall, text generally elicits fewer saccades. However, under the condition of “Co-text emojis,” the number of fixations on the text is even less than when it is used alone. In fact, the linear model did not reveal any interaction effects. The main effect of frequency is not significant, and only the main effect of the usage mode is significant.

Figure 3 shows the proportion of different saccade behaviors within the area of interest in the total number under different conditions. It can be seen that under the conditions of Pro-text emojis,the proportions of saccade numbers reach 15 and 25% respectively; under the conditions of only text, the proportions of saccade behaviors are 32 and 31%, respectively. Under the Co-text emojis conditions, there are certain proportions of participants skipping text and skipping emojis, and the proportions of various behaviors vary at different frequencies.

## 4 Discussion

In this research endeavor, Chinese sentences were utilized as the Experimental stimulis, and the Total Fixation Duration (TFD), First Fixation Duration (FFD), and Number of Saccades in the Area of Interest (NSA) of the target region served as the key metrics to investigate the role of emoji usage frequency and functions in the process of recognition and comprehension. The TFD, on the other hand, represents an overarching index of word recognition, encapsulating the entire process of identifying a word. Among these metrics, the FFD is particularly sensitive to the initial processing of lexical items. It reflects the early stage operations involved in word recognition, during which preliminary phonetic, orthographic, and potentially semantic information is gleaned. For instance, the visual presentation form of a lexical item serves as another core input in the early-stage processing. Within the time window corresponding to FFD (typically 100–200 milliseconds), the brain prioritizes the extraction of the basic features of orthography, rather than conducting refined detailed analysis of it. The purpose of this preliminary parsing is to quickly match the orthography of the current lexical item with the lexical representation library stored in the brain, and determine whether it conforms to orthographic rules. The acquisition of semantic information during the FFD stage exhibits the characteristics of “potentiality.” That is to say, the brain does not achieve complete semantic comprehension at this stage; instead, it makes a preliminary prediction of the lexical item’s semantic category based on the already acquired phonetic and orthographic cues. In reading or visual cognition research, a saccade refers to the rapid movement of the eyes from one fixation point to another. The number of saccades in the area of interest denotes the frequency of rapid eye movements within a specified region, reflecting two core aspects of cognitive processing: higher saccade counts often signify more intricate cognitive engagement with stimuli, such as when participants encounter challenging text or complex visuals and make repeated saccades to gather detailed information for brain analysis and comprehension; additionally, saccade frequency within an AOI reveals patterns of attention allocation, where frequent saccades indicate sustained or repeated focus on content within that region, typically driven by the presence of salient, critical, or cognitively demanding information requiring iterative examination.

### 4.1 The influence of frequency on the processing of Emojis

The experimental findings of this study clearly indicate that the usage frequency of emojis exerts a significant influence on both the FFD and TFD. However, it has no influence on the number of saccades. Specifically, a higher usage frequency corresponds to shorter FFD and TFD values for emojis. This result underscores the importance of frequency as a critical variable in lexical recognition, aligning with the outcomes of previous research.

The E-Z Reader model (Reichle et al., 1998) posits that word frequency is a pivotal factor in lexical recognition. Our experimental results are in accordance with the assumptions of this model. In the early processing stage, there is no significant difference in the FFD between high frequency emojis and words. This could potentially be attributed to the use of non-facial emojis in our experiment; these emojis are more visually intuitive, thereby facilitating faster processing. The visual features of high frequency emojis may be more typical and representative, making them easily and quickly captured and processed by the visual system. In addition, due to frequent exposure, readers have developed certain memories and perception habits regarding the visual patterns of high frequency emojis, enabling them to carry out visual encoding and recognition more efficiently. On the other hand,more visual searching and analysis are required to complete the recognition, which in turn affects the first fixation duration.

Furthermore,the total fixation time for low frequency Pro -text emojis is 200 ms longer than that for high frequency ones. This indicates that the processing stage for low frequency emojis takes longer. We postulate that another contributing factor to the longer TFD of low frequency emojis is the lack of semantic standardization. When participants encounter such emojis, there may not be a well defined, socially agreed upon meaning readily accessible in their mental lexicon. Consequently, deducing the meaning solely from the visual features of these emojis can be challenging (Miller et al., 2016). In contrast, Pro-text emojis have achieved lexicalization through long-term usage, developing fixed semantic orientations. In this regard, emojis behave just like standard words.

In the experimental results, the Number of Saccades in AOI is not affected by the usage frequency of emojis, this indicates that in the late processing stage, the frequency of emojis has no impact on lexical processing. On the one hand, this may be related to the stable cognitive patterns of native Chinese speakers. People will form a relatively fixed cognitive pattern when reading. For symbols with graphic integrity, such as Chinese characters and emojis, readers tend to perceive and process them as a whole. This cognitive pattern will not be easily changed by the usage frequency of emojis. Regardless of how frequently emojis appear, readers will identify them as a whole in a similar way. On the other hand, the bias of reading strategies towards text processing may also be a factor contributing to this result, readers often adopt relatively stable reading strategies during the reading process. For example, when reading content that includes emojis and texts, readers may first focus on the text to obtain the main information and use emojis as Supplementary Information to understand the emotions of the text or supplement details. Therefore, the NSA is relatively stable and will not change significantly with the increase or decrease of the frequency.

### 4.2 The influence of functions on the processing of emojis

The different results of the three eye movement indicators under the various experimental conditions provide empirical support for our exploration of the high frequency Pro -text emojis are lexicalized emojis. We observed that when an Pro -text emojis in a Chinese sentence, it generally elicits a longer fixation duration compared to a word in the same semantic context. Conversely, Co -text emojis total fixation duration is shorter than that of the word. The underlying reasons for these phenomena are analyzed in the subsequent subsections. This may be because different usage patterns lead to inconsistent roles of emojis in the text, which is caused by different processing mechanisms.

In the early-stage recognition and processing during sentence comprehension, participant showed a relatively longer first fixation duration for the Pro-text emojis, but exhibited the same recognition speed as that for words when it came to the Co-text emojis. We surmise that this could be due to the unpredictable appearance of non-facial emojis in text. This rarity likely captures participants’ attention. Additionally, the pictorial nature of emojis inherently renders them more attention grabbing than plain text, consistent with the findings of Pieters and Wedel (2004). Pro-text emojis pose greater challenges in integration compared to either the words or Co-text emojis. One plausible explanation is the infrequency of such usage, as indicated by the responses of our experimental participants in the survey. Irrespective of an emoji’s familiarity, when emojis participate in the semantic and syntactic integration of sentences, the low predictability of this usage pattern (Hale, 2001) demands heightened attention and more intricate cognitive processing.

In addition, high frequency Pro-text emojis have a slightly longer FFD than Chinese characters(18 ms). After in-depth thinking, we believe that this phenomenon is highly likely to be related to the special experimental materials with Chinese as the stimulus in this experiment. Looking back at the relevant research achievements, in an influential study conducted by Barach et al. (2021), it was clearly proposed that emojis demonstrate a much stronger ability to attract the attention of the audience compared to ordinary text. However, there is no significant difference in the first fixation duration between frequently used Pro-text emojis and words. This may be because, due to their frequent appearance in daily communication, people are already very familiar with the meanings and functions of frequently used Pro-text emojis, just as they are familiar with commonly used words. Such a high level of familiarity enables the brain to quickly recognize and understand them when processing, without the need to spend additional time deciphering their meanings. As a result, the processing time during the first fixation is similar to that of words.

In the integration stage, high frequency Pro-text emojis require a longer total fixation time (50 ms) than Chinese characters, while the total fixation time for Co-text emojis is shorter than that for characters. To determine whether the differences in reading times between emojis and their corresponding words were solely due to readers’ surprise upon encountering emojis, which could potentially confound the experimental results, we conducted an additional analysis. We compared the reading times of the stimulus materials at the start and end of the experiment. If surprise were the sole factor, we would expect this effect to dissipate over the course of the experiment. Contrary to our expectations, the pattern of experimental results (longest reading time for Pro-text emojis, followed by Co-text emojis, and shortest for words) remained consistent throughout. Although reading times generally decreased in later trials, especially for emojis, this pattern was stable. This finding is consistent with the results of Potter et al. (1986), who made target words visually distinct in presented sentences. Their additional manipulation did not alter the overall response time pattern. We concur with Potter et al. (1986) and conclude that the observed effects are not mere artifacts of surprise but rather represent a distinct cognitive process.

Roman Jakobson (1960) proposed that when ideographic symbols appear, the sender converts them into text (i.e., linguistic form) through a specific code, and the text is then transmitted to the receiver via a medium. In contrast, the processing of Pro-text emojis takes longer precisely because they first need to be transformed into corresponding linguistic concepts, while text can be processed directly by the brain. This additional conversion step increases the overall processing time for Pro-text emojis. Unlike Paggio and Tse (2022), who used English (a non-logographic writing system) and observed significant differences(300 ms between the text and the Pro-text emojis) in processing between Pro-text emojis, Co-text emojis, and the text itself. In our study that used Chinese, there was only a 50 ms difference in TFD between high frequency Pro-text emojis and the corresponding text. The human brain processes emojis may be similar to the interpretation of ideographic scripts such as Chinese characters. Chinese writing is logographic, which emojis have also been claimed to be, and the brain may employ some similar neural cognitive mechanisms when parsing these two types of visual symbols.

In terms of experimental trends, our results are generally consistent with those reported by Cohn et al. (2018) regarding the reading times of Pro-text emojis. They found that emojis are read approximately 50% slower than their corresponding words, whereas we observed relatively faster processing speeds for emojis. It is important to note that the reading times reported by Cohn et al. are substantially longer than those measured in our study. They reported an average reading time of over 1,100 ms for common words and approximately 2 s for emojis. In our view, these self- paced reading times may overestimate natural reading speeds. The extended fixation times for individual words and emojis in their study could be attributed to differences in task execution methods.

The fact that the total fixation time of Co-text emojis is shorter than that of Chinese characters precisely explains why emojis are so popular on the Internet nowadays. For one thing, reading habits play a role. Native Chinese speakers have developed a text centric reading strategy over time. They are highly proficient in processing and integrating text information, which has become an automated process. When emojis are mixed with text, readers instinctively prioritize text processing, treating emojis as visual adjuncts that can be quickly scanned for meaning, thereby reducing the need for prolonged fixation. Secondly, Co-text emojis accompanied by text have shorter processing times because they are influenced by the priming effect of orthographic words that appear directly before them.

In the experiment, extremely few NSA were detected for low frequency emojis. This is likely because, for the subjects, when processing these Pro-text emojis used as substitutes, they must first convert them into corresponding linguistic concepts and then integrate this information with the text. Owing to the infrequent use of these emojis, the subjects are less familiar with them and thus cannot quickly grasp their meanings. As a consequence, it is challenging for the subjects to skip over these emojis. They need to carefully process these symbols to ensure the integrity of the sentence’s semantic content. If the relevant noun concept is absent, the semantic information of the sentence will be incomplete. Furthermore, within the textual context of the experiment, emojis function as ideographic symbols replacing words. The context demands that the subjects understand the meaning of these symbols to correctly interpret the entire sentence. Skipping an emoji would lead to the loss of information regarding a specific noun in the sentence, thereby disrupting the coherence and integrity of the context. Hence, the context requirements prevent the subjects from easily bypassing these emojis. They must convert the emoji code into a linguistic code to comprehend its meaning and integrate it with the text.

As indicated by the NSA metric, there is no significant difference between the usage of emojis and text, suggesting certain similarities. This can be attributed to two main factors. Firstly, similar to Chinese text, emojis lack explicit word segmentation cues. Chinese readers rely on semantic and lexical knowledge to demarcate reading units, and the same is true for emojis, which are visually continuous with adjacent emojis or text and require readers to make independent distinctions. Secondly, both Chinese characters and emojis possess graphic integrity. Chinese characters, as square shaped ideograms, represent relatively independent meaning units. Emojis, presented as graphical symbols, also have strong integrity, typically conveying a specific emotion, concept, or object. They are perceived as a whole during reading, similar to Chinese characters, unlike English words which are composed of linearly arranged letters. However, further research is needed to determine whether emoji processing is more similar to that of Chinese characters than to English.

## 5 Conclusion

This study focuses on the processing of emojis, particularly that of high frequency Pro-text emojis. Using Chinese sentences as stimuli, it explores the processing pathways of emojis and the influencing mechanisms of usage frequency and their functions in sentences on this process through an eye-tracking experiment. The experimental results reveal three core patterns in emoji processing.

First, usage frequency exerts a significant impact on both the early and late stages of processing. High frequency emojis exhibit shorter First Fixation Duration (FFD) and Total Fixation Duration (TFD), enabling rapid recognition and efficient integration similar to that of words. In contrast, low-frequency emojis require longer processing time due to insufficient semantic standardization. Second, the linguistic functions of emojis regulate processing mechanisms. For Pro-text emojis, their low usage frequency leads to longer fixation durations, increasing attention demands and integration difficulty. However, for Co-text emojis, the FFD in the early processing stage is comparable to that of words, and the TFD in the integration stage is shorter—this characteristic aligns with readers’ reading habits and holistic cognitive patterns. Third, the Number of Saccades in the Area of Interest (NSA) is not affected by frequency, with no significant difference between emojis and text in this indicator. This stability stems from Chinese readers’ fixed holistic processing pattern of graphic symbols and text-centric reading strategy, reflecting the inherent similarity between emojis and text in visual-semantic processing. This echoes the previous conclusion that “the similarity between emojis and text in terms of NSA originates from graphic integrity and word segmentation characteristics.”

This study confirms that emojis are gradually integrating into daily communication, and the processing of high frequency Pro-text emojis is similar to that of standard words. More importantly, it verifies that high frequency Pro-text emojis in the Chinese context have achieved lexicalization, thus validating the theory of emoji lexicalization proposed by Storment (2024). Usage frequency and the functions of emojis in sentences jointly influence emoji processing: high frequency emojis gain advantages in recognition and integration, while Pro-text emojis and Co-text emojis alter their roles in text through differences in their functional positions.

Methodologically, using Chinese as the experimental material provides a new perspective for the study. Chinese possesses a significant semantic preview advantage and shares compact spatial-semantic features with emojis, offering a unique entry point for research on symbol processing. Meanwhile, the application of eye-tracking technology to conduct refined analysis of the early FFD and late TFD processing stages enriches the empirical data on emoji lexical access.

This study has certain limitations. First, visual features (such as complexity and saturation) were not standardized. da Silva et al. (2024) found that colors significantly influence the decoding of emotions in emojis, highlighting that unstandardized visual features like color could introduce confounding variables in emoji processing research. Specifically, Liao et al. (2022) demonstrated that color significantly affects the recognition of emoticon expressions, highlighting that unstandardized visual features like color could introduce confounding variables in emoji processing research. However, how to balance the visual features of Pro-text emojis— which we use as linguistic units in daily communication—remains a challenge. Second, the experimental materials were limited to Chinese, which restricts the generalizability of the conclusions—the unique processing pattern of logographic scripts may differ from that of alphabetic languages relying on linear letter sequencing. Third, the static laboratory-based reading paradigm is inconsistent with the dynamic, multimodal environment of real social media, resulting in limited ecological validity.

Future research can be improved in the following aspects. First, tools like EMO-DB can be used to standardize visual features, thereby isolating frequency effects from semantic effects. Second, cross-linguistic comparisons can be expanded to alphabetic and other languages to clarify the universal symbolic mechanisms and language-specific characteristics of emoji processing. Third, mobile eye-tracking technology can be adopted to construct ecological paradigms, capture real-time processing in natural digital communication, and reveal the laws of contextual variability and multisensory integration. Addressing the aforementioned limitations will further enhance the validity and generalizability of the conclusions, facilitating in-depth exploration of the evolving role of emojis in language communication.

## Data Availability

The raw data supporting the conclusions of this article will be made available by the authors, without undue reservation.

## References

[ref1] Barach E. Feldman L. B. Sheridan H. (2021). Are emojis processed like words?: eye movements reveal the time course of semantic processing for emojified text. Psychon. Bull. Rev. 28, 978–991. doi: 10.3758/s13423-020-01864-y, PMID: 33511541

[ref2] Christofalos A. Feldman L. Sheridan H. (2022). “Semantic congruency facilitates memory for emojis.” in *Proceedings of the 5th international workshop on emoji understanding and applications in social media*. pp.63–68.

[ref3] Cohn N. (2016). A multimodal parallel architecture: a cognitive framework for multimodal interactions. Cognition 146, 304–323. doi: 10.1016/j.cognition.2015.10.007, PMID: 26491835

[ref4] Cohn N. Engelen J. Schilperoord J. (2019). The grammar of emoji? Constraints on communicative pictorial sequencing. Cogn. Res. Princip. Implicat. 4, 1–18. doi: 10.1186/s41235-019-0177-0, PMID: 31471857 PMC6717234

[ref5] Cohn N. Roijackers T. Schaap R. Engelen J. (2018). “Are emoji a poor substitute for words? Sentence processing with emoji substitutions*.” Proceedings of the 40th Annual Conference of the Cognitive Science Society*, pp. 1524–1529

[ref6] Cohn N. Schilperoord J. (2022). Remarks on multimodality: grammatical interactions in the parallel architecture. Front. Artif. Intell. 4:778060. doi: 10.3389/frai.2021.778060, PMID: 35059636 PMC8764459

[ref7] da Silva J. F. F. Moura W. M. de M. Paradeda R. B. (2024). “Decoding Emotions: The Influence of Colors on Emojis.” In *Proceedings of the XVI Brazilian Symposium on Ubiquitous and Pervasive Computing (SBCUP 2024) (11–20)*. Porto Alegre: Brazilian Computer Society.

[ref8] Ganis G. Kosslyn S. M. Stose S. Thompson W. L. Yurgelun-Todd D. A. (1996). Neural correlates of category and modality specificity of visual recognition memory: a PET study. NeuroImage 4, 237–247.

[ref9] Gustafsson V. (2017). Replacing words with emojis and its effect on reading time. Umeå’s 21st Student Conference in Computing Science, Umeå, Sweden.

[ref10] Hale J. (2001). “A probabilistic earley parser as a psycholinguistic model.” in *Proceedings of NAACL*.

[ref11] Hancock P. M. Hilverman C. Cook S. W. Halvorson K. M. (2024). Emoji as gesture in digital communication: Emoji improve comprehension of indirect speech. Psychon. Bull. Rev. 31, 1335–1347. doi: 10.3758/s13423-023-02411-1 38010455

[ref12] Hintz F. Khoe Y. H. Strauß A. Psomakas A. J. A. Holler J. (2023). Electrophysiological evidence for the enhancement of gesture-speech integration by linguistic predictability during multimodal discourse comprehension. Cogn. Affect. Behav. Neurosci. 23, 340–353. doi: 10.3758/s13415-023-01074-8, PMID: 36823247 PMC9949912

[ref13] Holler J. Levinson S. C. (2019). Multimodal language processing in human communication. Trends Cogn. Sci. 23, 465–478. doi: 10.1016/j.tics.2019.05.006 31235320

[ref14] Jakobson R. (1960). Closing statement: linguistics and poetics. Style in language

[ref15] Kelly R. Watts L. (2015). Characterising the inventive appropriation of emoji as relationally meaningful in mediated close personal relationships. Experiences of technology appropriation: Unanticipated users, usage, circumstances, and design, Oslo, Norway

[ref16] Liao S. Sakata K. Paramei G. V. (2022). Color affects the recognition of emoticon expressions. I-Perception 13:20416695221080778. doi: 10.1177/20416695221080778, PMID: 35265312 PMC8900290

[ref17] Lo S. K. (2008). The nonverbal communication functions of emoticons in computer-mediated communication. CyberPsychol. Behav. 11, 595–597. doi: 10.1089/cpb.2007.0132, PMID: 18817486

[ref18] Miller H. Thebault-Spieker J. Chang S. Johnson I. Terveen L. Hecht B. (2016). “Blissfully happy” or “ready to fight”: varying interpretations of emoji. In *Proceedings of the international AAAI conference on web and social media* (10, 1, pp. 259–268).

[ref19] Neel L. A. G. Mckechnie J. G. Robus C. M. Hand C. J. (2023). Emoji alter the perception of emotion in affectively neutral text messages. J. Nonverbal Behav. 47, 83–97. doi: 10.1007/s10919-022-00421-6

[ref20] Ousterhout J. (2017). Electrophysiological evidence for semantic processing of emojis in written language. Neuropsychologia 104, 360–367. doi: 10.1016/j.neuropsychologia.2017.07.018

[ref21] Paggio P. Tse A. P. P. (2022). Are emoji processed like words? An eye-tracking study. Cogn. Sci. 46:e13099. doi: 10.1111/cogs.13099, PMID: 35122294

[ref22] Pérez A. Schmidt E. Kourtzi Z. Tsimpli I. (2020). Multimodal semantic revision during inferential processing: the role of inhibitory control in text and picture comprehension. Neuropsychologia 138:107313. doi: 10.1016/j.neuropsychologia.2019.107313, PMID: 31904356

[ref23] Pierini Francesco (2021). “Emojis and gestures: a new typology.” *Proceedings of Sinn Und Bedeutung* 25, pp. 720–732.

[ref24] Pieters R. Wedel M. (2004). Attention capture and transfer in advertising: brand, pictorial, and t. J. Mark. 68, 36–50. doi: 10.1509/jmkg.68.2.36.27794

[ref25] Potter M. C. Kroll J. F. Yachzel B. Carpenter E. Sherman J. (1986). Pictures in sentences: understanding without words. J. Exp. Psychol. Gen. 115, 281–294. doi: 10.1037/0096-3445.115.3.281, PMID: 2944988

[ref26] Prada M. Rodrigues D. L. Garrido M. V. Lopes D. Cavalheiro B. Gaspar R. (2018). Motives, frequency and attitudes toward emoji and emoticon use. Telematics Inform. 35, 1925–1934. doi: 10.1016/j.tele.2018.06.005

[ref27] Reichle E. D. Pollatsek A. Fisher D. L. Rayner K. (1998). Toward a model of eye movement control in reading. Psychol. Rev. 105, 125–157. doi: 10.1037/0033-295X.105.1.125, PMID: 9450374

[ref28] Robus C. M. Hand C. J. Filik R. Pitchford M. (2020). Investigating effects of emoji on neutral narrative text: evidence from eye movements and perceived emotional valence. Comput. Hum. Behav. 109:106361. doi: 10.1016/j.chb.2020.106361

[ref29] Rodrigues D. L. Cavalheiro B. P. Prada M. (2022). Emoji as icebreakers? Emoji can signal distinct intentions in first-time online interactions. Telematics Inform. 69:101783. doi: 10.1016/j.tele.2022.101783

[ref30] Scheffler T. Brandt L. de la Fuente M. Nenchev I. (2022). The processing of emoji-word substitutions: a self- paced-reading study. Comput. Hum. Behav. 127:107076. doi: 10.1016/j.chb.2021.107076 PMC9240971 35781978

[ref31] Schlenker P. (2019). Gestural semantics: replicating the typology of linguistic inferences with pro-and post-speech gestures. Nat. Lang. Linguist. Theory 37, 735–784. doi: 10.1007/s11049-018-9414-3

[ref9001] Schotter E. R. Angele B. Rayner K. (2012). Parafoveal processing in reading. Attention, Perception, & Psychophysics, 74, 5–35. 10.3758/s13414-011-0219-2 22042596

[ref32] Scott G. G. O’Donnell P. J. Leuthold H. Sereno S. C. (2009). Early emotion word processing: evidence from event-related potentials. Biol. Psychol. 80, 95–104. doi: 10.1016/j.biopsycho.2008.03.010 18440691

[ref9002] Spinks J. A. Gao J. H. Tan L. H. Liu H. L. Fox P. T. Perfetti C. A. (2001). The neural system underlying Chinese logograph reading. Academic Press. 10.1006/nimg.2001.0749 11304080

[ref33] Storment J. D. (2024). Going lexicon? The linguistic status of pro-text emojis. Glossa J. Gen. Linguist. 9, 1–43. doi: 10.16995/glossa.10449

[ref34] Tang M. Chen B. Zhao X. Zhao L. (2020). Processing network emojis in Chinese sentence context: an ERP study. Neurosci. Lett. 722:134815. doi: 10.1016/j.neulet.2020.134815, PMID: 32027951

[ref35] Tieu L. Schlenker P. Chemla E. (2019). Linguistic inferences without words. Proc. Natl. Acad. Sci. USA 116, 9796–9801. doi: 10.1073/pnas.1821018116, PMID: 31019076 PMC6525514

[ref36] Weissman B. (2019). “Emojis in sentence processing: an electrophysiological approach.” W*WW’19: companion proceedings of the 2019 world wide web conference*, pp. 478–479.

[ref37] Yan M. Richter E. M. Shu H. Kliegl R. (2009). Readers of Chinese extract semantic information from parafoveal words. Psychon. Bull. Rev. 16, 561–566. doi: 10.3758/PBR.16.3.561, PMID: 19451385

[ref38] Yang J. Wang S. Tong X. Rayner K. (2012). Semantic and plausibility effects on preview benefit during eye fixations in Chinese reading. Read. Writ. 25, 1031–1052. doi: 10.1007/s11145-010-9281-8, PMID: 22593624 PMC3337412

