# Supplementary material for: The lexicalization of emojis: the influence of frequency and functions of emojis in sentences on this process—a study based on eye movement tracking
Source: Front Psychol. 2025 Sep 17;16:1631967. doi: 10.3389/fpsyg.2025.1631967 (PMC12484118; doi:10.3389/fpsyg.2025.1631967)
Supplement: Supplementary file 1 [file Table_1.DOCX]

# Appendix A: Questionnaire

调查问卷

一 基本信息

1. 您的年龄
2. 您的性别
3. 您的眼睛状况（是否近视/散光）
4. 您使用哪种手机系统（iOS/安卓/其他/我不知道）
5. 您日常使用emoji的频率（从不/偶尔/经常）
6. 七度量表
7. 判断以下emoji意义的可接受度（不可接受1，非常接受5）
8. 你觉得“
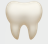
🎈”的意思是“牙齿”吗？
9. 你觉得“
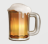
🎈”的意思是“啤酒”吗？
10. 你觉得“
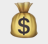
🎈”的意思是“金钱”吗？
11. 你觉得“
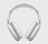
🎈”的意思是“耳机”吗？
12. 你觉得“
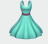
🎈”的意思是“裙子”吗？
13. 你觉得“
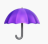
🎈”的意思是“雨伞”吗？
14. 你觉得“
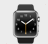
🎈”的意思是“手表”吗？
15. 你觉得“
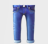
🎈”的意思是“裤子”吗？
16. 你觉得“
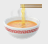
🎈”的意思是“面条”吗？
17. 你觉得“
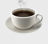
🎈”的意思是“咖啡”吗？
18. 你觉得“
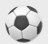
🎈”的意思是“足球”吗？
19. 你觉得“
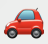
🎈”的意思是“汽车”吗？
20. 你觉得“
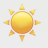
🎈”的意思是“太阳”吗？
21. 你觉得“
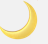
🎈”的意思是“月亮”吗？
22. 你觉得“
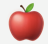
🎈”的意思是“苹果”吗？
23. 你觉得“
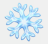
🎈”的意思是“雪花”吗？
24. 你觉得“
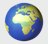
🎈”的意思是“地球”吗？
25. 你觉得“
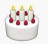
🎈”的意思是“蛋糕”吗？
26. 你觉得“
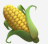
🎈”的意思是“玉米”吗？
27. 你觉得“
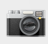
🎈”的意思是“相机”吗？
28. 判断以下句子的可接受度（不可接受1，非常接受5）
29. 这杯🍺
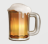
很醇厚。
30. 这个
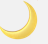
很皎洁。
31. 那个
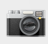
很小巧。
32. 这个
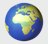
很美丽。
33. 这片
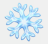
很特别。
34. 那天
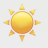
很耀眼。
35. 这根
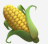
很香甜。
36. 这辆
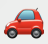
很节能。
37. 这杯
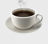
很浓郁。
38. 这颗
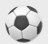
很耐用。
39. 那袋
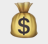
很充裕。
40. 这款
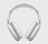
很智能。
41. 这颗
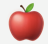
很好吃。
42. 那块
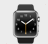
很时尚。
43. 这条
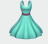
很简约。
44. 这把
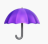
很小巧。
45. 这碗
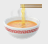
很劲道。
46. 这颗
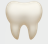
很脆弱。
47. 那条
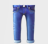
很百搭。
48. 那天
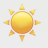
很耀眼。
49. 判断以下emoji的使用频率（不使用1，最常使用5）

（1）
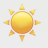


（2）
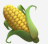


（3）
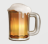


（4）
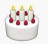


（5）
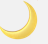


（6）
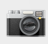


（7）
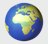


（8）
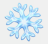


（9）
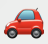


（10）
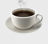


（11）
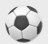


（12）
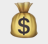


（13）
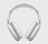


（14）
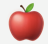


（15）
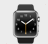


（16）
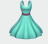


（17）
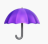


（18）
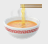


（19）
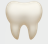


（20）
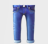


# Appendix B: Stimuli experiment

1. 那天太阳很耀眼。

那天
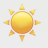
很耀眼。

那天太阳
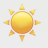
很耀眼。

1. 这杯啤酒很醇厚。

这杯🍺
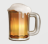
很醇厚。

这杯啤酒
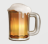
🍺很醇厚。

1. 那个蛋糕很诱人。

那个
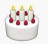
很诱人。

那个蛋糕
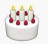
很诱人。

1. 这个月亮很皎洁。

这个
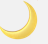
很皎洁。

这个月亮
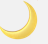
很皎洁。

1. 那个相机很小巧。

那个
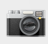
很小巧。

那个相机
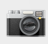
很小巧。

1. 这个地球很美丽。

这个
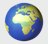
很美丽。

这个地球
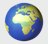
很美丽。

1. 这片雪花很特别。

这片
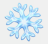
很特别。

这片雪花
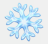
很特别。

1. 这辆汽车很节能。

这辆
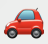
很节能。

这辆汽车
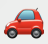
很节能。

1. 这杯咖啡很浓郁。

这杯
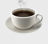
很浓郁。

这杯咖啡
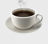
很浓郁。

1. 这颗足球很耐用。

这颗
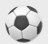
很耐用。

这颗足球
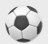
很耐用。

1. 那袋金钱很充裕。

那袋
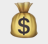
很充裕。

那袋金钱
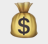
很充裕。

1. 这款耳机很智能。

这款
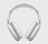
很智能。

这款耳机
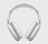
很智能。

1. 这颗苹果很好吃。

这颗
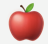
很好吃。

这颗苹果
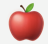
很好吃。

1. 那块手表很时尚。

那块
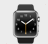
很时尚。

那块手表
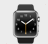
很时尚。

1. 这条裙子很简约。

这条
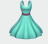
很简约。

这条裙子
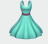
很简约。

1. 这把雨伞很小巧。

这把
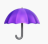
很小巧。

这把雨伞
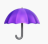
很小巧。

1. 这碗面条很劲道。

这碗
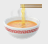
很劲道。

这碗面条
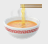
很劲道。

1. 这颗牙齿很脆弱。

这颗
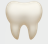
很脆弱。

这颗牙齿
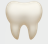
很脆弱。

1. 那条裤子很百搭。

那条
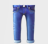
很百搭。

那条裤子
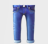
很百搭。

1. 这根玉米很香甜。

这根
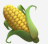
很香甜。

这根玉米
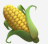
很香甜。

# Appendix C: Fillers experiment

1. 这个汉堡很美味。
2. 那朵玫瑰很娇嫩。
3. 这栋房子很古老。
4. 那个气球很独特。
5. 这颗药丸很刺鼻。
6. 这款口红很畅销。
7. 那只小狗很可爱。
8. 那枚戒指很精致。
9. 这架飞机很安全。
10. 那只老鼠很讨厌。
11. 那道彩虹很绚丽。
12. 那片云朵很柔软。
13. 那辆单车很轻便。
14. 这块西瓜很多汁。
15. 这包香烟很便宜。
16. 这个手机很流畅。
17. 这颗柠檬很酸爽。
18. 这个奖杯很珍贵。
19. 这顶帽子很合适。
20. 这块饼干很可口。

# Appendix A: Questionnaire

questionnaire

I. Basic information

1. Your age
2. Your gender
3. Your eye condition (whether you are nearsighted or astigmatic)
4. What mobile operating system do you use (iOS/Android/Other/Dont know)
5. How often you use emojis on a daily basis (never/occasionally/frequently)
6. The seven-point scale
7. Judge the acceptability of the meaning of the following emoji (unacceptable 1, very acceptable 5)
8. You think🎈 Does "🎈" mean "tooth"?
9. You think🎈 Does "🎈" mean "beer"?
10. You think🎈 Does "money" mean "money"?
11. You think"🎈 Does "🎈" mean "headphones"?
12. You think🎈 Does "it" mean "skirt"?
13. You think🎈 Does "🎈" mean "umbrella"?
14. You think🎈 Does "🎈" mean "watch"?
15. You think🎈 Does "🎈" mean "pants"?
16. You think🎈 Does "noodles" mean "面条"?
17. You think"🎈 Does "🎈" mean "coffee"?
18. You think"🎈 Does "football" mean "football"?
19. You think🎈 Does "car" mean "car"?
20. You think🎈 Does "meaning" mean "sun"?
21. You think"🎈 Does "🎈" mean "moon"?
22. You think🎈 Does "Apple" mean "apple"?
23. You think🎈 Does "snowflake" mean "snowflake"?
24. You think🎈 Does "Earth" mean "Earth"?
25. You think🎈 Does "cake" mean "cake"?
26. You think"🎈 Does "corn" mean "corn"?
27. You think"🎈 Does "camera" mean "camera"?
28. Judge the acceptability of the following sentences (unacceptable 1, very acceptable 5)
29. This is the 🍺Its very rich.
30. this oneIts very pure.
31. thatSmall.
32. this onevery beautiful 。
33. This landIts special.
34. that dayIts brilliant.
35. This oneIts sweet.
36. This oneIts very energy efficient.
37. This oneIts very rich.
38. This oneVery durable.
39. That bagIts very plentiful.
40. this typeIts smart.
41. This oneyummy 。
42. That blockIts very fashionable.
43. This oneIts very simple.
44. This is itSmall size.
45. This bowlIts very strong.
46. This oneIts very fragile.
47. That oneIts very versatile.
48. that dayIts brilliant.
49. Judge the frequency of use of the following emoji (use 1 if not, use 5 if most)

（1）

（2）

（3）

（4）

（5）

（6）

（7）

（8）

（9）

（10）

（11）

（12）

（13）

（14）

（15）

（16）

（17）

（18）

（19）

（20）

# Appendix B: Stimuli experiment

1. The sun was blazing that day.

that dayIts brilliant.

The sun that dayIts brilliant.

1. This beer is very mellow.

This is the 🍺Its very rich.

This beer🍺 Its very rich.

1. That cake looks so tempting.

thatIts tempting.

That cakeIts tempting.

1. The moon was very bright.

this oneIts very pure.

This moonIts very clean.

1. The camera was tiny.

thatSmall.

That cameraSmall.

1. The earth is beautiful.

this onevery beautiful 。

This planetvery beautiful 。

1. This snowflake is special.

This landIts special.

This snowflakeIts special.

1. This car is very energy efficient.

This oneIts very energy efficient.

This carIts very energy efficient.

1. This coffee is very strong.

This oneIts very rich.

This cup of coffeeIts very rich.

1. This football is very durable.

This oneVery durable.

This footballVery durable.

1. That bag of money was abundant.

That bagIts very plentiful.

That bag of moneyIts very plentiful.

1. This headset is smart.

this typeIts smart.

This earphoneIts smart.

1. This apple is delicious.

This oneyummy 。

This appleyummy 。

1. That watch is very fashionable.

That blockIts very fashionable.

That watchIts very fashionable.

1. This dress is very simple.

This oneIts very simple.

This dressIts very simple.

1. This umbrella is very small.

This is itSmall.

This umbrellaSmall.

1. This bowl of noodles is very strong.

This bowlIts very strong.

This bowl of noodlesIts very strong.

1. This tooth is fragile.

This oneIts very fragile.

This toothIts very fragile.

1. That pair of trousers is very versatile.

That oneIts very versatile.

That pair of trousersIts very versatile.

1. This corn is very sweet.

This oneIts sweet.

This cornIts sweet.

# Appendix C: Fillers experiment

1. This burger is delicious.
2. The rose was delicate.
3. The house is very old.
4. That balloon was unique.
5. This pill is very pungent.
6. This lipstick is very popular.
7. The puppy was lovely.
8. That ring is exquisite.
9. The plane was safe.
10. The rat was revolting.
11. The rainbow was gorgeous.
12. The clouds were soft.
13. The bike was very light.
14. This watermelon is very juicy.
15. The pack of cigarettes was very cheap.
16. This phone is smooth.
17. This lemon is very sour.
18. This trophy is very precious.
19. The hat fits perfectly.
20. This biscuit is delicious.
